# Comparison of Homocysteine-Lowering Drugs

**DOI:** 10.1371/journal.pmed.0020145

**Published:** 2005-05-31

A link between high levels of homocysteine, a sulfur-containing amino acid, and heart disease was first suggested in the 1960s, when it became clear that patients with inborn errors of homocysteine metabolism were prone to develop severe cardiovascular disease in their teens and twenties. Treatment with homocysteine-lowering substances such as folate, vitamin B12, and betaine reduces the incidence of heart attacks and strokes in these patients.

This led to the hypothesis that mildly elevated levels of homocysteine might contribute to vascular disease. Subsequently, several studies have found higher mean homocysteine levels in patients with coronary, peripheral, and cerebral vascular disease, particularly in those with vascular disease not readily explained by conventional risk factors such as high low-density lipoprotein (LDL) cholesterol, diabetes, or smoking. Several studies then sought to determine whether elevated homocysteine levels were a cause or effect of cardiovascular disease, and evidence for a causal relationship is accumulating. However, whether reduction in homocysteine levels translates into a reduction in heart disease is still an open question.

“We are keenly awaiting the results from several ongoing trials. In the meantime, our group is trying to determine the risks and benefits associated with different homocysteine-lowering nutrients,” said Margreet Olthof. She and her colleagues at the Wageningen Centre for Food Science analyzed four independent, placebo-controlled, randomized intervention studies that examined the effects of betaine, folic acid, and phosphatidylcholine on plasma homocysteine concentrations in healthy volunteers. They combined blood lipid data from the individual studies and compared changes in blood lipid concentrations between individuals taking homocysteine-lowering nutrients and those taking placebo.

They found that that betaine supplementation, while effective at lowering homocysteine, also increased LDL cholesterol and triacylglycerol.

This raises the possibility that any potential benefits for cardiovascular health would be undermined by the adverse effects on blood lipids, and make betaine less suitable as a homocysteine-lowering agent in healthy individuals. The data on phosphatidylcholine were inconclusive, but supplementation of folic acid—the most common way to lower homocysteine levels—does not seem to affect blood lipids. The researchers conclude that folic acid “therefore remains the preferred treatment for lowering of blood homocysteine concentrations” in healthy individuals.

